# Environmental factors associated with incidence of developmental dysplasia of the hip: a systematic review and meta-analysis

**DOI:** 10.1186/s12891-023-07073-7

**Published:** 2023-12-05

**Authors:** Yu-Yi Huang, Wei-Chun Lee, Chia-Hsieh Chang, Wen-E Yang, Hsuan-Kai Kao

**Affiliations:** 1 Department of Orthopedic Surgery, Chang Gung Memorial Hospital, Keelung, Taiwan; 2 Department of Pediatric Orthopaedics, Chang Gung Memorial Hospital, Taoyuan, Taiwan; 3 College of Medicine, Chang Gung University, Taoyuan, Taiwan

**Keywords:** DDH, Temperature, Associated factor, Screening

## Abstract

**Background:**

Established associated factors for DDH include female sex, breech presentation, family history, congenital malformations, oligohydramnios, and maternal hyperthyroidism. However, evidence for environmental factors that may contribute to DDH is limited and inconsistent.

**Methods:**

A systematic review of medical literature was conducted to collect data on environmental factors, including latitude, longitude, average yearly precipitation, average yearly temperature, minimum monthly temperature, and maximum monthly temperature, from all institutions that published articles on DDH. Univariate linear regression analysis was used to examine the correlation between environmental factors and DDH incidence, while multiple regression analysis was conducted to identify significant associated factors for DDH incidence.

**Results:**

Data from a total of 93 unique manuscripts were analyzed, revealing a significant negative correlation between DDH incidence and temperature, including average yearly temperature (r = -0.27, p = 0.008), minimum monthly temperature (r = -0.28, p = 0.006), and maximum monthly temperature (r = -0.23, p = 0.029). Additionally, there was a significant positive correlation between DDH incidence and latitude (r = 0.27, p = 0.009), and a significant negative correlation between DDH incidence and average yearly precipitation (r = -0.29, p = 0.004). In the final multiple regression analysis, temperature, including average yearly temperature, minimum monthly temperature, and maximum monthly temperature, were identified as significant associated factors for DDH incidence.

**Conclusion:**

The findings of this study suggest an association between cold weather and DDH incidence. Further research should explore the link between cold weather and DDH incidence, offering insights into potential interventions for cold climates.

## Background

Developmental dysplasia of the hip (DDH) represents a prevalent musculoskeletal disorder in children, with significant clinical implications if not detected and managed early [1]. Delayed diagnosis of DDH often necessitates surgical interventions, such as open reduction and femoral or pelvic osteotomy for hip dysplasia or dislocation, and in severe cases, even total hip replacement due to early development of osteoarthritis [2]. Given these potentially life-altering consequences, the early identification of DDH and the implementation of hip screening programs for infants at risk are established norms in neonatal healthcare practices worldwide [3–5].

Numerous studies and a substantial body of literature have examined various factors associated with DDH, encompassing well-established risk factors like birth order, female gender, breech presentation, family history of hip dysplasia, congenital malformations, oligohydramnios, and maternal hyperthyroidism [1, 4, 6, 7]. Although there is evidence suggesting the involvement of intrinsic factors such as hormones, nutrition, and genetic markers based on research in canines, the application of these findings to humans remains speculative [8, 9]. The limited and inconsistent evidence surrounding these potential risk factors highlights the complexity of DDH etiology [10].

Furthermore, extrinsic factors, including swaddling practices, tight clothing, and the influence of environmental conditions like cold weather, have been proposed as possible contributors to DDH development [11–14]. Notably, the association between DDH and environmental factors, especially the occurrence of DDH cases during winter, has been a subject of interest. However, the intricate interplay between extrinsic and intrinsic factors complicates the ability to confirm whether winter is a significant, independent environmental factor influencing DDH incidence [4, 11, 12, 15].

Moreover, a limited number of studies have explored the incidence of DDH across different countries [12, 16, 17]. However, these findings may not provide a comprehensive understanding due to geographic constraints. Consequently, this study aims to redefine the relationship between DDH incidence and environmental factors by conducting a thorough analysis of evidence derived from a worldwide dataset. In doing so, we aim to offer insights into the complex interactions in DDH etiology.

## Materials and methods

We conducted a systematic review on DDH that focused on incidence, etiology, epidemiology, prevalence, and demographics. We excluded manuscripts that discussed the treatment of DDH, teratological DDH, or the incidence of DDH with congenital malformations. To ensure a comprehensive search, the keyword searched included the following MeSH terms “developmental dysplasia of the hip”, “DDH”, “congenital dysplasia of the hip”, and “CDH” combined with the Boolean operator “AND” and all synonyms combined with Boolean operator “OR”. We included non-English language articles, such as those in Chinese, Japanese, Korean, Russian, Spanish, Portuguese, and Italian. The databases we used for our search included PubMed/Medline and EMBASE. We also searched individual orthopedic journals, such as the Journal of Bone and Joint Surgery (American Volume and British Volume), Journal of Pediatric Orthopaedics, Clinical Orthopedic and Related Research, The Bone and Joint Journal, and Acta Orthopaedica Scandinavica. We checked on PROSPERO website and there was no pending meta-analysis on this topic. Regardless of the evidence levels of the literature, including ecological studies, prospective studies, retrospective studies, and case series, we included any publications that mentioned the incidence of DDH, whether diagnosed through physical examination, ultrasound, or radiography.

For this analysis, we used the geographic and weather parameters of the cities where the articles were published. The environmental factors we examined included latitude, longitude, average yearly precipitation, average yearly temperature, minimum monthly temperature, and maximum monthly temperature. We obtained the data for latitude and longitude from the National Geographic Atlas of the World [18]. We analyzed latitude values between 0–90° north latitude and 0–90° south latitude as positive. Similarly, we analyzed longitude values between 0-180° east longitude as positive and those between 0-180° west longitude as negative. We obtained average yearly precipitation, average yearly temperature, minimum monthly temperature, and maximum monthly temperature data from the National Climate Data Center [19].

To enhance the robustness of our statistical methodology, we have provided a more comprehensive explanation of our approach for managing missing data, addressing outliers, and dealing with potential multicollinearity within climatic variables.

1. Handling Missing Data: In our study, missing data was minimal due to the rigorous data collection process. Any missing data points were addressed by employing multiple imputation techniques, which allowed us to impute missing values based on observed data, thereby maintaining the integrity of the dataset.
2. Outlier Management: Robust statistical analyses require the identification and appropriate handling of outliers. Outliers were detected using the Tukey method and subsequently scrutinized to assess their impact on the results. Outliers that significantly deviated from the norm were considered influential observations and were subjected to sensitivity analysis to determine their effect on the overall findings.
3. Multicollinearity Mitigation: Given the interrelated nature of environmental variables, potential multicollinearity was addressed to ensure the validity of our results. We assessed multicollinearity among climatic variables using variance inflation factor (VIF) analysis. As VIF value for all the independent variable was less than 10, it suggested lower risks of multicollinearity.

Our statistical approach aimed to enhance the analysis of the relationship between environmental factors and DDH incidence. We followed established best practices in addressing missing data, managing outliers, and dealing with multicollinearity to uphold result reliability. All statistical analyses were conducted using SPSS software (IBM Corp, version 20.0, Armonk, New York), and the statistical significance threshold was set at *p* < 0.05 unless otherwise specified. This rigorous methodology strengthens the validity of our findings and ensures the integrity of the statistical analyses.

## Results

**Fig. 1:**
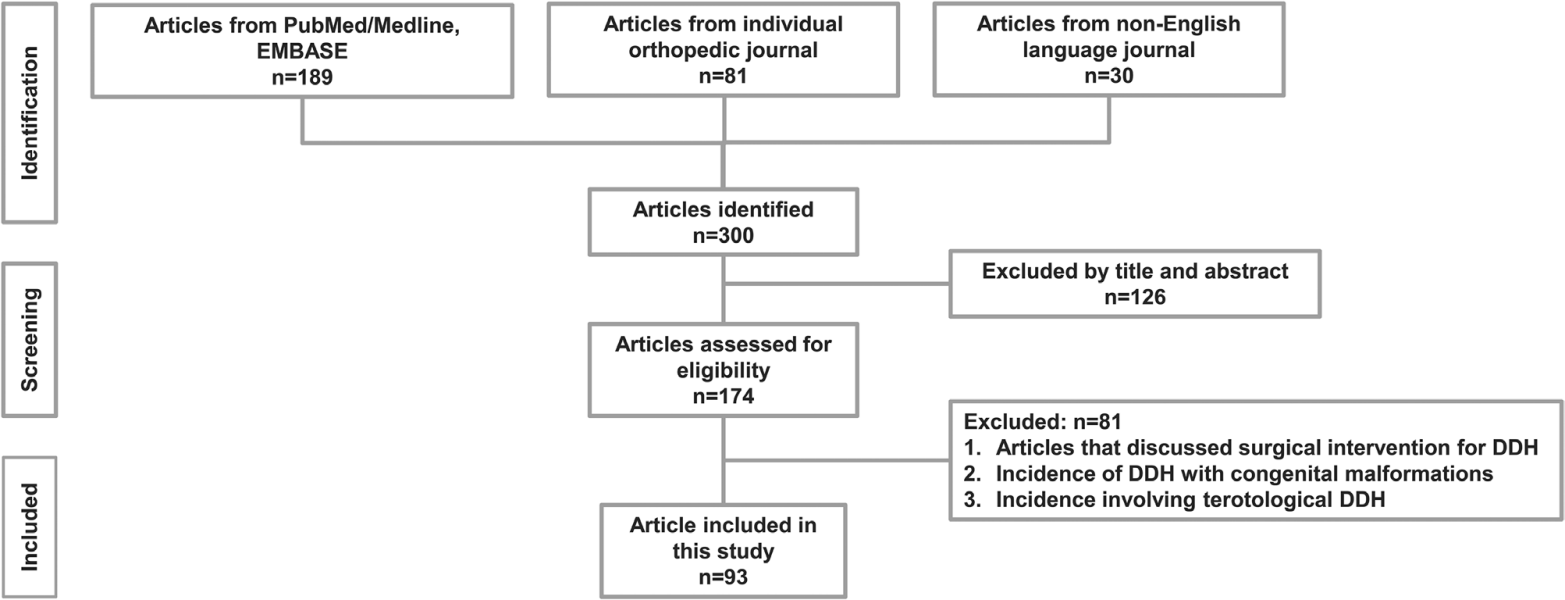
PRISMA diagram of study selection process

**Fig. 2:**
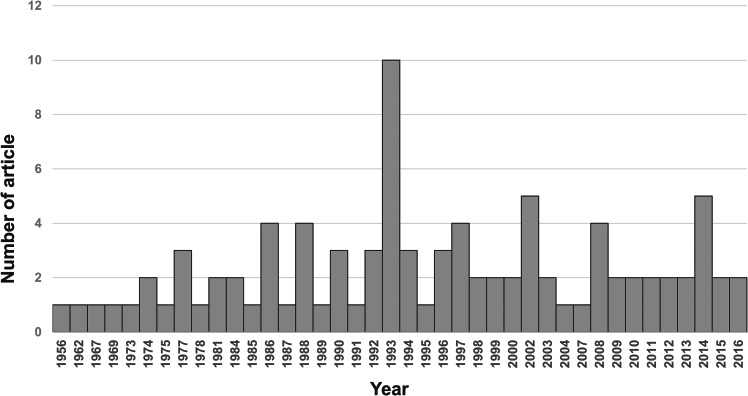
Distribution of eligible articles by publication year

Our search yielded 270 English and 30 non-English language articles. We excluded 126 articles due to ineligible abstracts and titles, leaving a total of 174 unique manuscripts for detailed review. Among these, 93 articles that provided ample information were included in this study. The flow diagram depicting the search and identification of articles is shown in (Fig. 1). The distribution of articles by publication year is shown in (Fig. 2). Specifically, there were 12 articles published before 1980, 18 articles from 1981 to 1990, 31 articles from 1991 to 2000, 17 articles from 2001 to 2010, and 15 articles published after 2010.

**Fig. 3:**
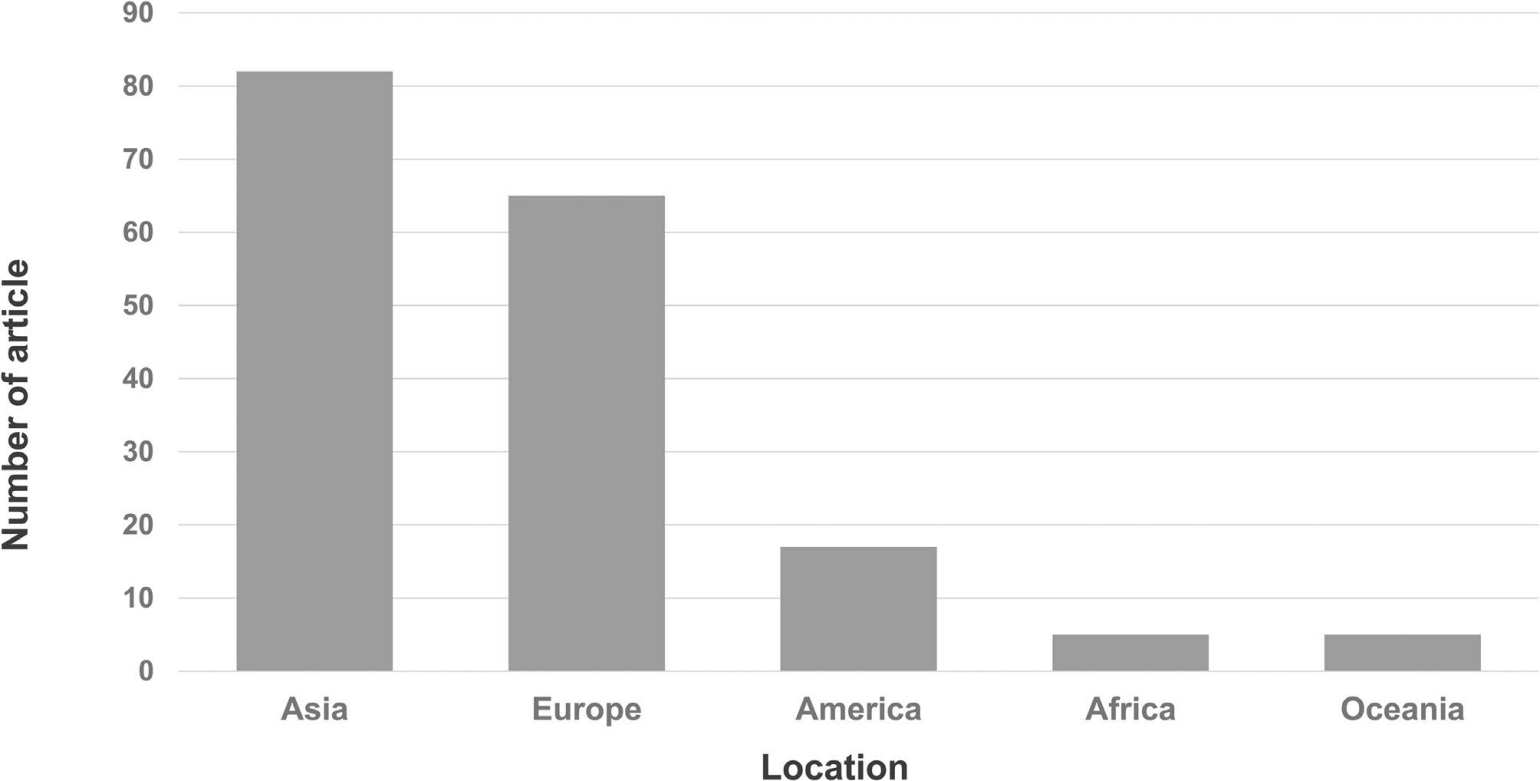
Distribution of eligible articles by geographic location

**Fig. 4:**
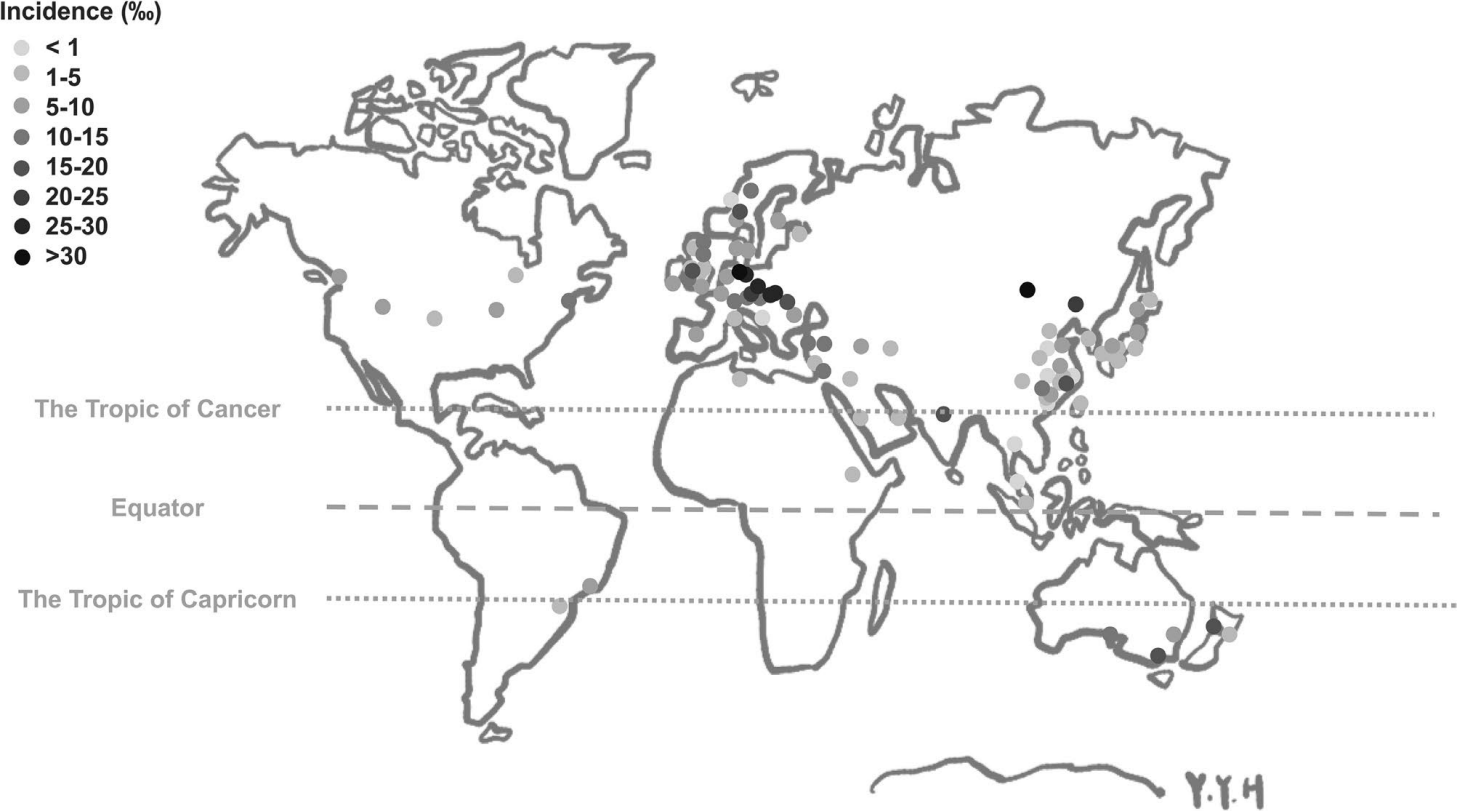
Global incidence of DDH depicted on a world map

The articles were distributed across different regions, with 43 from Asia (incidence‰: 6.0 ± 7.1), 35 from Europe (incidence‰: 11.3 ± 8.9), 6 from North America (incidence‰: 7.4 ± 3.9), 2 from Central/South America (incidence‰: 6.9 ± 2.6), 2 from Africa (incidence‰: 2.8 ± 1.8), and 5 from Oceania (incidence‰: 11.3 ± 6.4) (Fig. 3). However, there was no significant difference in the incidence of DDH among the five continents (Table 1). The incidence of DDH on five continents is also shown on a world map [18] (Fig. 4).

**Table 1:** Incidence of DDH among five continents

	Asia (N = 43)	Europe (N = 35)	North America (N = 6)	Central/South America (N = 2)	Africa (N = 2)	Oceania (N = 5)	*p-value
‡Incidence‰ Mean (SD)	6.0 (7.1)	11.3 (8.9)	7.4 (3.9)	6.9 (2.6)	2.8 (1.8)	11.3 (6.4)	0.06

‡ The data of incidence are mean, with standard deviation (SD) in parentheses

*Comparison incidence of DDH among five continents with use of ANOVA

**Fig. 5:**
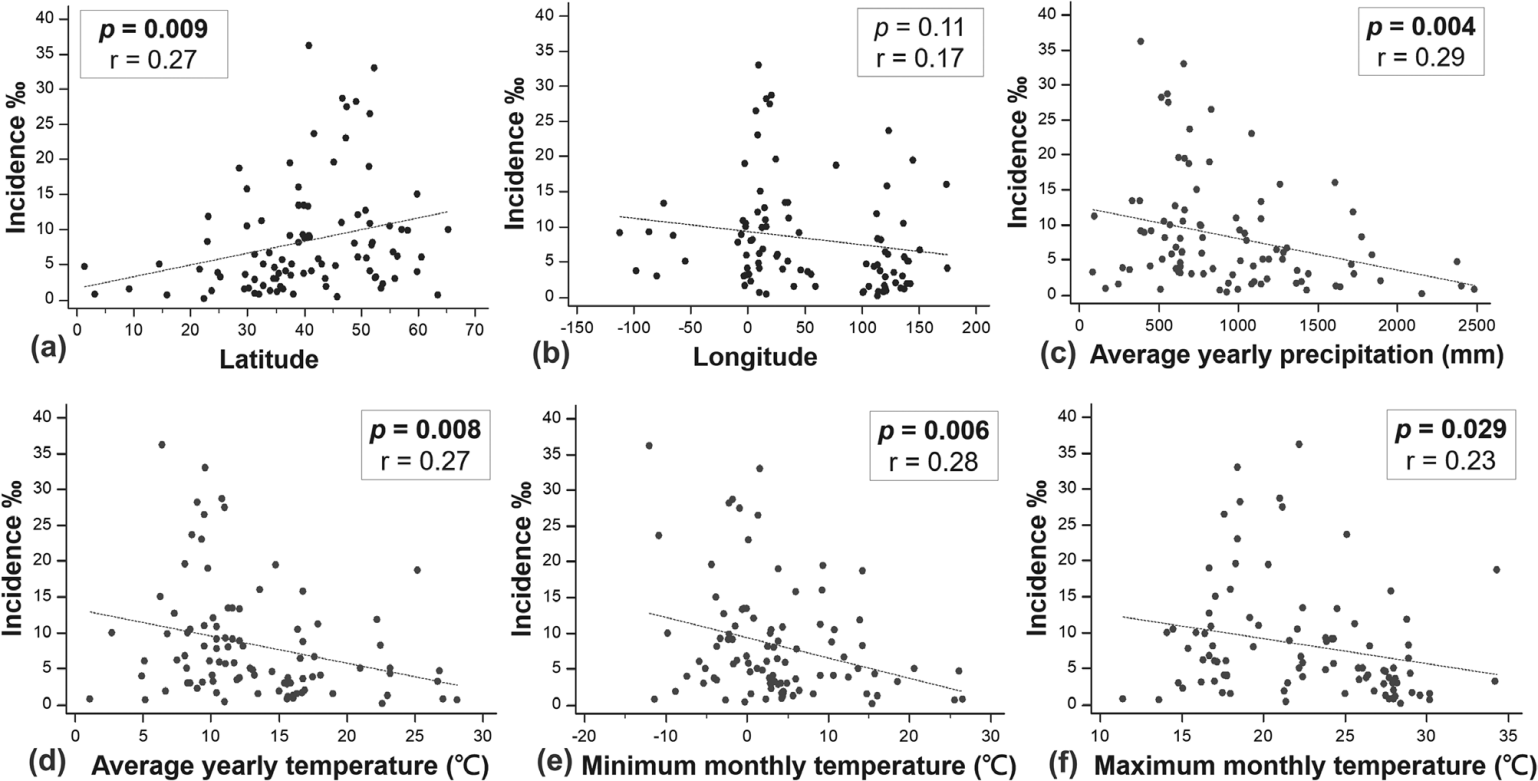
Univariate linear regression analysis for the incidence of DDH with the following environmental factors: **(a)** latitude **(b)** longitude **(c)** average yearly precipitation **(d)** average yearly temperature **(e)** minimum monthly temperature **(f)** maximum monthly temperature

The results of our univariate linear regression analysis indicated a significant positive correlation between the incidence of DDH and latitude (r = 0.27, *p* < 0.05). In addition, we found a significant negative correlation between the incidence of DDH and average yearly precipitation (r = − 0.29, *p* < 0.05), average yearly temperature (r = − 0.27, *p* < 0.05), minimum monthly temperature (r = − 0.28, *p* < 0.05), and maximum monthly temperature (r = − 0.23, *p* < 0.05). However, we did not find a significant correlation between the incidence of DDH and longitude (r = − 0.28, *p* = 0.11) (Table 2) (Fig. 5). Based on these results, we conducted a multiple regression analysis using the significant factors identified in the univariate analysis. The final analysis revealed that average yearly temperature, minimum monthly temperature, and maximum monthly temperature were significant factors associated with the incidence of DDH (Table 3).

**Table 2:** Factors affecting the incidence of DDH: univariate analysis

Study (n = 93)	Correlation coefficient (r)	*p*-value
Latitude	0.27	**0.009**
Longitude	-0.17	0.11
Average yearly precipitation	-0.29	**0.004**
Average yearly temperature	-0.27	**0.008**
Minimum monthly temperature	-0.28	**0.006**
Maximum monthly temperature	-0.23	**0.03**

Significant *p* values are shown in bold

**Table 3:** Factors affecting the incidence of DDH: multiple regression analysis

Independent variables	Coefficient	Odds ratio	95% CI	*p* - value
Latitude	0.19	0.90	0.78 − 0.15	0.14
Average yearly precipitation	-0.002	1.00	0.99–1.01	0.32
Average yearly temperature	-6.24	0.02	0.004–1.11	**0.003**
Minimum monthly temperature	-3.03	5.27	0.90–30.9	**0.002**
Maximum monthly temperature	-3.25	7.45	1.00-55.48	**0.003**

CI: confidence interval; significant *p* values are shown in bold

## Discussion

Our study found that the incidence of DDH is significantly higher in regions with lower average yearly temperature, minimum monthly temperature, or maximum monthly temperature. This finding is the most important result and is also the greatest predictor in our final multiple regression analysis. One possible explanation for these findings is related to the use of swaddling [20–22], which is still common in many countries with cold weather, despite long-term policies promoting newborn health care [13, 23]. Swaddling involves wrapping the baby in tighter clothing or blankets to protect them from the cold and promote uninterrupted sleep [20, 24]. However, this can lead to hip instability, as the tight clothing or swaddling increase extension and adduction of the hips, resulting in Ortolani-positive hip instability [16, 25]. In contrast, the incidence of DDH is lower in high-temperature regions such as Hong Kong, Bangkok, and Malawi, where back-carrying is used instead of swaddling [14, 26]. Back-carrying involves carrying the baby in a flexion, abduction position that is similar to the Pavlik harness, which is mostly used for DDH treatment [27]. Another hypothesis is related to abnormal levels of nutrients such as calcium, vitamin C, and vitamin D, which are associated with cold temperatures and correlated with joint laxity and delayed bone remodeling, potentially contributing to the development of DDH [9, 11].

The univariate analysis revealed a significant correlation between the incidence of DDH and latitude, but this was not found to be significant in the final multiple regression analysis. This may be due to the fact that even at approximately the same latitude, there can be different environmental factors, such as temperature, precipitation, humidity, and climate between two cities (for example, Mashhad and Takmaya). This heterogeneity of environmental factors can obscure the correlation between latitude and DDH incidence, leading to different patterns of variation.

Loder’s literature review [11] found variable patterns of DDH incidence with 70.3% showing a single winter peak, which only partially supported the association between tight clothing and cold temperatures with DDH. However, it’s important to note that our study didn’t specifically examine the link between DDH incidence and seasonal temperature fluctuations, resulting in different conclusions. This divergence highlights the multifaceted nature of DDH incidence, indicating that various factors, including the interaction of genetic, external, and internal elements, contribute to its complexity [11]. Similarly, Lee et al. [12] analyzed surgical treatment for late-diagnosed DDH from 1999 to 2010 in Taiwan. Their findings align with ours, emphasizing the importance of cold weather as an associated factor. Yet, due to Taiwan’s subtropical climate, generalizing this relationship to different regions may be challenging. In contrast, our study aimed to collect data from various geographic areas, making our findings more applicable across different regions.

The absence of data from continents like South America and Africa may be attributed to the lack of robust public health infrastructure for DDH prevention and diagnosis in these regions or other higher prevalence of congenital diseases that might overshadow DDH. Nevertheless, based on the literature we have gathered, it appears that DDH incidence is lower in countries with higher temperatures. It is plausible that the missing data from these South American and African nations contributes to our findings, indicating a weak correlation between DDH incidence and temperature. The limited sample from these countries could indeed influence the overall conclusions of this study.

Cold weather can pose health risks to the pediatric population globally, such as asthma exacerbations, allergic rhinitis, and atopic dermatitis [28]. Moreover, cold weather and reduced sunlight exposure can hinder the cutaneous synthesis of vitamin D in children, potentially leading to vitamin D deficiency [29]. This deficiency is a known risk factor for nutritional rickets, one of the most prevalent pediatric bone diseases worldwide [30]. To address this concern, regions with high latitudes and cold climates have established guidelines for vitamin D supplementation, particularly for infants. For example, in North America, infant formula is fortified with vitamin D, and additional vitamin D supplementation is recommended for children with limited sunlight exposure [31]. In light of our study’s findings suggesting a relationship between DDH and temperature, further research is needed to delve into the causal aspects of this association. It is plausible that experiences with vitamin D supplementation may offer valuable insights for developing public health policies aimed at preventing and screening for DDH.

Our study has several strengths and limitations. One of its strengths is that it includes many global studies on the incidence of DDH, allowing for investigation and consideration of associated factors without geographical limitations. Additionally, the large sample size in our study can be representative and provide insight into the environmental factors associated with the incidence of DDH.

Some limitations of the current study should be noted. First, the selection bias may exist, that only approximately 20% of the literature reviewed is from tropical or subtropical zones, with the remaining 80% from temperate zones. Second, the study did not include some important information, such as the definition of DDH, the age of the children, and the screening methods used. Additionally, the diagnosis criteria for DDH were not included in the analysis, which may have led to overestimation or underestimation of the incidence of DDH. Future studies should consider using consistent diagnosis criteria and surgical intervention information in a larger geographic area to further explore the relationship between environmental factors and the incidence of DDH.

## Conclusions

The findings of this study suggest an association between cold weather and DDH incidence, emphasizing the importance of further exploration in this field. Future research endeavors could delve into the mechanisms underlying this connection, consider the influence of cultural practices, and explore potential interventions for regions prone to colder climates. This work opens the door to a promising avenue for understanding DDH, and we encourage subsequent studies to build upon these findings to contribute to our knowledge of this complex condition.

## Data Availability

The datasets used and analysed during the current study available from the corresponding author on reasonable request.

## List of abbreviations

DDH: developmental dysplasia of the hip
